# Identification of the sex chromosome system in a sand fly species, *Lutzomyia longipalpis* s.l

**DOI:** 10.1093/g3journal/jkab217

**Published:** 2021-06-26

**Authors:** Felipe M Vigoder, Luciana O Araripe, Antonio Bernardo Carvalho

**Affiliations:** 1 Departamento de Genética, Instituto de Biologia, Universidade Federal do Rio de Janeiro, CCS, Rio de Janeiro sl A2-075 21941-971, Brazil; 2 Laboratório de Biologia Molecular de Insetos, Instituto Oswaldo Cruz, FIOCRUZ, Rio de Janeiro, Brazil

**Keywords:** sand flies, sex chromosome system, Y chromosome, YGS, *Lutzomyia longipalpis*, Psychodidae: Diptera, Genetics of Sex

## Abstract

In many animal species, sex determination is accomplished by heterogamety *i.e.*, one of the sexes produces two types of gametes, which upon fertilization will direct the development toward males or females. Both male (“XY”) and female (“ZW”) heterogamety are known to occur and can be easily distinguished when the sex-chromosomes are morphologically different. However, this approach fails in cases of homomorphic sex chromosomes, such as the sand fly *Lutzomyia longipalpis s.l.* (Psychodidae, Diptera), which is the main vector of visceral leishmaniosis in Brazil. In order to identify the heterogametic sex in *L. longipalpis* s.l., we did a whole-genome sequencing of males and females separately and used the “Y chromosome Genome Scan” (YGS) method to find sex-specific sequences. Our results, which were confirmed by PCR, show that *L. longipalpis* s.l. has XY system. The YGS method can be especially useful in situations in which no morphological difference is observed in the sex-chromosomes or when fresh specimens are not readily available.

## Introduction

Sex determination is a major biological phenomenon with important practical and theoretical consequences. In many animal species, sex determination is accomplished by genes in specialized chromosomes, called sex-chromosomes (White 1973; Bull 1983; Rice 1996; Charlesworth 2006; Bachtrog *et al.* 2011; Blackmon *et al.* 2017). Sex chromosomes are often heteromorphic and are called XY system when males are the heterogametic sex and ZW when females have the heterogametic chromosome (Bull 1983; Blackmon *et al.* 2017). Most Diptera have heteromorphic sex chromosome systems (mainly XY) (Kaiser and Bachtrog 2010; Ashman *et al.* 2014; Vicoso and Bachtrog 2015; Blackmon *et al.* 2017) but other systems occur like in sand flies (Psychodidae; Diptera) where the sex chromosomes are homomorphic (Kreutzer *et al.* 1987, 1988). The commonness of heteromorphic XY systems in Diptera might suggest that this is the ancestral state, but actually there were numerous replacements of both the X and the Y chromosomes (Vicoso and Bachtrog 2015; Dupim *et al.* 2018). The search for sex determination systems and sex determination genes in Diptera and insects in general is an active area of research, and an important phenomena remain to be elucidated (Hall *et al.* 2015; Krzywinska *et al.* 2016; Sharma *et al.* 2017).

The standard theory of the evolution of a new sex chromosome invokes the acquisition of a sex-determining locus by an ordinary autosome. Sexually antagonistic selection then favors the suppression of recombination around the associated sex-determining region, which may extend to the whole chromosome (Charlesworth 2017). The lack of recombination will cause an increase in genomic divergence between the homologs with and without the sex-determining locus, and the chromosome associated with the heterogametic sex gradually degenerates. This process will ultimately lead to the formation of morphologically different (heteromorphic) sex chromosomes, making it easy to tell them apart. XY systems originated when the sex-determining locus determines maleness (“M factor”), whereas ZW systems originated from a female determining gene factor (“F factor”) (Charlesworth 1991; Kaiser and Bachtrog 2010; Wright *et al.* 2016; Furman *et al.* 2020). The degree of differentiation is a continuum across species from full homology (which is expected in very young systems) to nearly full differentiation (*e.g.*, mammals, birds); it is assumed to correlate with the time of divergence, but there are numerous exceptions (Charlesworth and Charlesworth 2005; Palmer *et al.* 2019; Furman *et al.* 2020).

Sex chromosome systems are generally stable, but transitions across groups are more common than previously thought (Carvalho and Clark 2005; Kaiser and Bachtrog 2010; Bachtrog *et al.* 2011; Ashman *et al.* 2014; Blackmon and Demuth 2015; Vicoso and Bachtrog 2015; Abbott *et al.* 2017; Blackmon *et al.* 2017; Dupim *et al.* 2018; Jeffries *et al.* 2018; Pennell *et al.* 2018). The classical approach to determine the sex chromosome system (XY *vs* ZW) is to observe the chromosomes during meiosis or mitosis in males and females. However, when there are no morphological differences between the sex chromosomes (homomorphy), cytogenetics cannot determine which system (XY or ZW) is present in a given organism. This is the case of most sand fly species studied so far, from both the *Phlebotomus* and *Lutzomyia* genera,(Kreutzer *et al.* 1987, 1988). The exceptions are *Phlebotomus perniciosus* and *Lutzomyia shannoni*, in which heteromorphic chromosomes were observed (Kreutzer *et al.* 1987; Jiménez *et al.* 2001). In both cases, the authors considered the heteromorphic pair as an XY pair, however, neither work was able to determine the sex of the larvae before dissection (Kreutzer *et al.* 1987; Jiménez *et al.* 2001), and hence these species may have as well a ZW system. As mentioned, despite the fact that most Diptera have an XY system, ZW species occur (Kaiser and Bachtrog 2010; Ashman et al. 2014; Vicoso and Bachtrog 2015; Blackmon *et al.* 2017). Therefore, the sex determination system of sand flies remains an open question.

*Lutzomyia longipalpis* s.l. (2N = 8) (Kreutzer *et al.* 1987) is the main vector of visceral leishmaniosis in Brazil. It actually is a complex of cryptic species (Souza *et al.* 2008, 2017; Araki *et al.* 2009, 2013; Vigoder *et al.* 2015), and identifying sex-specific sequences in *L. longipalpis* s.l. may provide important data about the evolutionary history of this group. It may also provide important tools for vector control strategies, especially in regard to genetic manipulation (Achee *et al.* 2019).

Recent bioinformatics methods have been used to identify the sex chromosome system, however, most rely on producing many genetic lines through crossing and/or expensive sequencing of DNA and/or RNA from many individuals (Tomaszkiewicz *et al.* 2017; Palmer *et al.* 2019). In order to identify the heterogametic sex, we adapted the “Y chromosome Genome Scan” (YGS) method developed by Carvalho and Clark (2013). This method uses separate sequencing from male and female DNA, followed by *de novo* assembly, and computational identification of sex-specific sequences. YGS was originally used to detect Y chromosome sequences in organisms with a known XY system. Since we do not know the sex-determination system of *L. longipalpis* s.l., we ran the YGS pipeline twice, assuming either a XY or a ZW system. The rational is that only one of these runs will detect sex-specific sequences; searching for a W in a XY system or searching for a Y in a ZW system should fail to retrieve any sequences.

## Material and methods

### Sand flies and DNA extraction

Sand flies were obtained from Dr. Fernando Genta (Laboratório de Fisiologia de Insetos Vetores, FIOCRUZ, RJ). The colony was founded over 30 years ago from flies collected at the locality of Jacobina (Bahia State, Brazil; 11° 11’ S, 40° 32’ W) with multiple reintroductions from the same population throughout the years. Larvae at L4 stadium were collected from rearing cups and individually put in *eppendorf* vials for pupation, to ensure that all emerging adults were virgins. Emerged adults were sexed and pooled into groups of 50 males and 50 females and preserved in −20°C ethanol. DNA extraction was performed with the Puregene DNA kit (Qiagen) following the manufacturer’s recommendations.

### DNA sequencing and assembly

A draft genome of *L. longipalpis* s.l. available in VectorBase (vectorbase.org/) was obtained from male individuals from Jacobina (LlonJ1_VB). However, for our analysis, we needed sequences from males and females separately and hence we did them as follows. Illumina DNA sequencing (100 bp paired-end; fragment size 470 bp) was performed by Macrogen (South Korea), with a HiSeq 2000 machine. A total of 4.4 Gb of data was produced for each sex. The raw coverage was ∼ 20× fold for each sex, as estimated by GenomeScope (Vurture *et al.* 2017). Illumina reads were trimmed using TrimGalore-0.5.0 (https://www.bioinformatics.babraham.ac.uk/projects/trim_galore/) and *de novo* assembled with SPADES v. 3.11.1 (Bankevich *et al.* 2012) using reads from both sexes. Scaffolds smaller than 200 bp were removed. Contaminants were detected with the Blobtools pipeline (Laetsch and Blaxter 2017), and scaffolds with hits with Bacteria in both BlastX and BlastN were also removed. The completeness of the assembly was evaluated using BUSCO v3 (Simão *et al.* 2015) using a dataset of 2799 conserved genes that are present in almost all available Diptera genomes (database name: diptera_odb9).

### YGS method

The YGS method was developed by Carvalho and Clark (2013) to computationally identify sex-specific sequences. Note that these sequences should be present only in the heterogametic sex (males in XY systems, or females in ZW), and hence YGS can be used to identify the sex determination system. Briefly, the YGS method starts with a genome assembly using sequences from the heterogametic sex or from both sexes, in order to have all chromosomes represented. Next, the assembled genome is decomposed into *k-mers* (we used 16 bp *k*-mers) and the repetitive *k*-mers (*i.e.*, those occurring more than once in the assembled genome) are identified. This step aims to avoid the interference of repetitive sequences such as transposable elements, which can be shared between, say, a Y chromosome and an autosome. The unassembled trimmed reads from the homogametic sex were also decomposed into *k*-*mers*. Finally, each scaffold is individually decomposed into *k*-mers, and the repetitive ones (identified as described above) are removed; this result in a set of single-copy *k*-mers, which was compared with the set of *k*-mers present in the reads of the homogametic sex. Scaffolds with a high percentage of unmatched *k*-mers are likely to belong to the Y (or W) chromosome, which can be experimentally confirmed with PCR for details Carvalho and Clark (2013). Because we did not know which sex of *L. longipalpis* s.l. is heterogametic (and hence has the sex-specific sequences) we performed the YGS twice, using the same mixed sex assembly and either males’ or females’ reads for comparison. Note that scaffolds coming from the autosomes and from the X or Z are expected to have a very low (or zero) proportion of unmatched *k*-*mers*. when compared with reads from males or from females.

**Figure 1:**
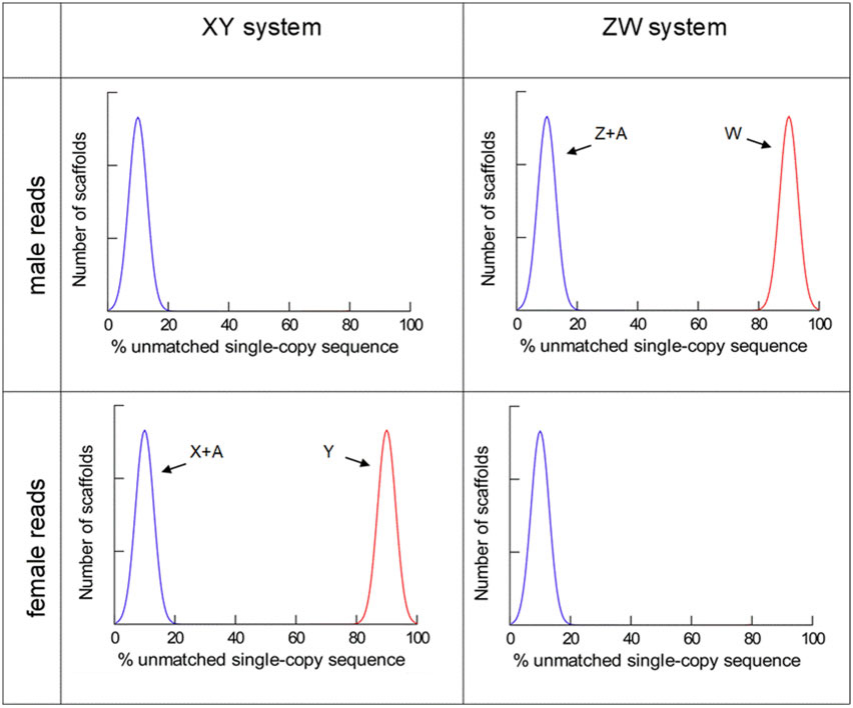
Identification of the sex-determining system using the YGS method. A genome assembled with both sexes was compared to male and female short reads, and the percentage of unmatched sequence of each scaffold is calculated. Blue: scaffolds which are present in both sexes (autosomes, and X or Z chromosomes); red: scaffolds that are present only in the heterogametic sex (Y or W chromosome). Note that the pattern of the distributions reveals the sex-determining system. We represented both peaks with the same height (*i.e.*, containing the same number of scaffolds), but in real cases they can be very different, pending on the relative amount of sequence on Y or W chromosomes, and their fragmentation in the assembly [see Figure 2 of Carvalho and Clark (2013) for real examples]. The peaks are not always centered in 0 and 100% due to assembly errors and other factors (Carvalho and Clark 2013).

On the other hand, scaffolds that came from the Y or W chromosome will have a high proportion of unmatched *k-mers* when compared against the reads of the homogametic sex (females in the case of Y, males in the case of W), but not when compared against the heterogametic sex. Hence, as shown in Figure 1, the sex-determination system is revealed by examining at the pattern of the distribution of unmatched sequences.

If the sex-determination system is male heterogamety (*i.e.*,a XY system), the proportion of unmatched *k-mers* should have a bimodal distribution when we use the reads from females, and a unimodal distribution when we use the reads from the males (Figure 1, left panels); the opposite pattern is should occur with female heterogamety (a ZW system; Figure 1, right panels).

### YGS in a ZW system

The YGS method had been only used in organisms with a XY system (Carvalho and Clark 2013; Dupim *et al.* 2018). As a proof of principle that it can work on an ZW system we used reads of males and females of *Tephritis californica* (Diptera: Tephritidae) from Vicoso and Bachtrog (2015) (SRR1738208 and SRR1738207, respectively). All the procedures used in the *L. longipalpis* s.l. analysis was used with *T. californica*, starting with the *de novo* assembly with SPAdes (see above).

### Data availability

The raw reads and *de novo* genome assembly of *L. longipalpis* s.l. is available in Bioproject PRJNA699015. Supplementary material is available at figshare: https://doi.org/10.25387/g3.14794824.

## Results

As a preliminary assessment of the quality of our genome assembly, we compared it with the one from VectorBase. Our *de novo* assembly of *L. longipalpis* s.l. has 39,920 scaffolds with a total of 148,058,403 bp and a N50 of 14,820 (Table 1). The VectorBase genome, in contrast, has only 11,532 “scaffolds,” a total size of 154,229,266, and N50 of 85,244. Hence, the VectorBase assembly is more contiguous, probably due to the use of long-range mate-pair libraries. However, BUSCO indicated that our assembly is more complete than VectorBase's both in terms of complete genes (82 *vs* 77%, respectively) and of missing genes (4.9 *vs* 16.6%, respectively; Table 2).

**Table 1:** **Assembly statistics for our *de novo* assembly and the genome available at VectorBase (LlonJ1;** https://www.vectorbase.org/organisms/lutzomyia-longipalpis/jacobina/llonj1)

Statistics without reference	*Lu. longipalpis*	LlonJ1 VB
# contigs	39.92	11.456
# contigs (≥0 bp)	39.92	11.532
# contigs (≥1,000 bp)	16.684	11.315
# contigs (≥5,000 bp)	7.074	2.813
# contigs (≥10,000 bp)	3.925	2.175
# contigs (≥25,000 bp)	1.121	1.467
# contigs (≥50,000 bp)	259	900
Largest contig	223.406	887.685
Total length	148.058.403	154.199.576
Total length (≥0 bp)	148.058.403	154.229.266
Total length (≥1,000 bp)	134.675.297	154.097.547
Total length (≥5,000 bp)	114.036.555	140.416.275
Total length (≥10,000 bp)	91.611.366	135.838.255
Total length (≥25,000 bp)	48.051.351	124.278.145
Total length (≥50,000 bp)	18.720.614	103.711.641
N50	14.82	85.244
N75	5.581	36.074
L50	2.484	490
L75	6.508	1.18
GC (%)	34.55	35
**Mismatches**		
# N's	384.752	11.457.932
# N's per 100 kbp	259.87	7.431

**Table 2:** BUSCO summary, showing the completeness of our *assembly (L. longipalpis*) and the genome available at VectorBase (LlonJ1_VB)

	*L. longipalpis*		LlonJ1_VB	
	*N*	%	*N*	%
Complete	2,290	81.8	2,156	77.0
Complete and single-copy	2,276	81.3	2,035	72.7
Complete and duplicated	14	0.5	121	4.3
Fragmented	373	13.3	179	6.4
Missing	136	4.9	464	16.6
Total groups searched	2,799		2,799	

**Figure 2:**
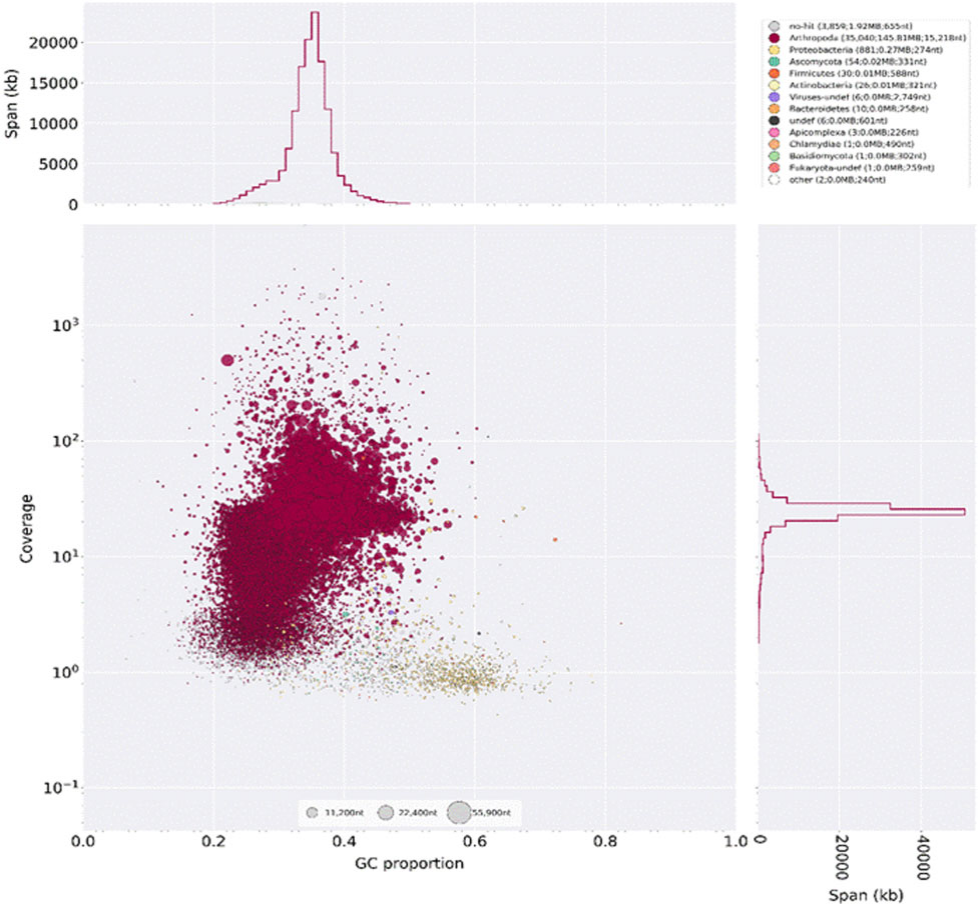
Blobtools plot showing the level of contamination of the genome. The circles represent genomic scaffolds. The size of the circles indicates scaffold size and the colors correspond to the taxonomic annotation based on BLASTn. The *x*-axis is the proportion of GC sequence and the *y*-axis represents genome coverage. Note that bacterial contaminants (yellow) have lower coverage and tend to have higher GC content.

The analysis of contaminants was done using the Blobtools pipeline (Simão *et al.* 2015). We performed both BlastN and BlastX to assign scaffolds to their likely origin (Arthropod, Bacteria, and so on). Scaffolds that had hits for bacteria, protozoa, and/or virus in both BlastN and BlastX were deemed as contaminants and removed. Note that this is a quite stringent criteria (*e.g.*, contaminant scaffolds containing only noncoding regions will not be flagged); we adopted it to avoid discarding real *L. longipalpis* s.l. sequences. Six hundred and fifty-seven scaffolds (amounting to 215,133 bp) were deemed as contaminants and were removed resulting in a final assembly of 147,843,270 bp. Figure 2 shows the result obtained with Blobtools using BlastN.

### Identification of sex-specific sequences

In order to identify the sex chromosome system of *L. longipalpis* s.l. we ran the YGS method twice on the same mixed-sex assembly. In the first run, we compared the assembly with female reads (a procedure which we will refer to as XY genome scan, or “XY-GS,” aiming to detect Y-linked contigs), and in the second run, we compared it to male reads (“ZW-GS,” aiming to detect W-linked contigs) (Supplementary File S1). In both cases, we removed from the analysis contigs that have low quality (6163 contigs, totaling 2,279,222 bp), defined as having more than 10% of their *k*-mers absent from the male and female reads. These spurious *k*-mers came from assembly errors.

**Figure 3:**
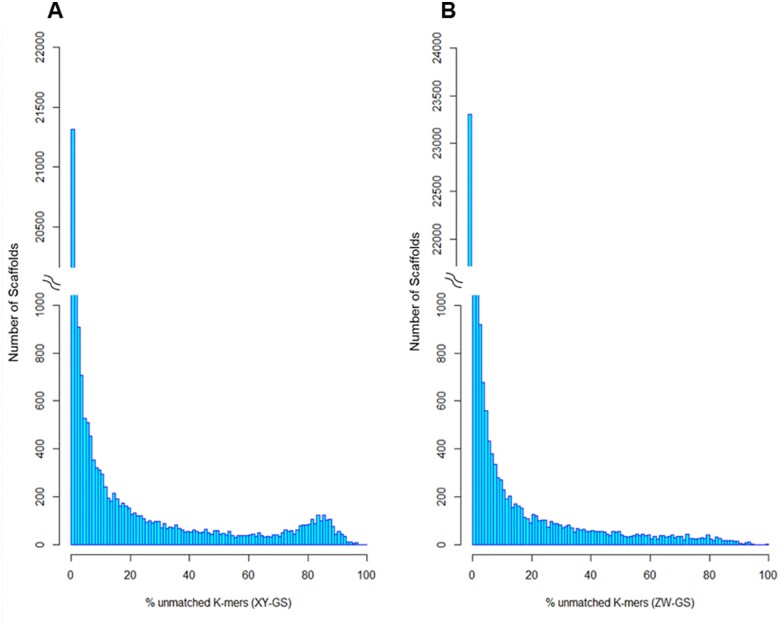
YGS results. The plot shows the distribution of scaffolds in relation to the proportion of valid-single copy unmatched sequences when either (A) the XY-GS or (B) the ZW-GS were used.

**Figure 4:**
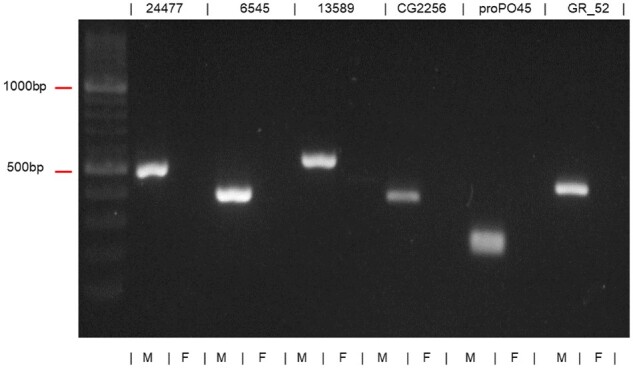
PCR gel of the candidate Y-linked sequences, confirming their male-specificity. Sequence names are shown on the top, DNA sample sex male (M) or female (F) is shown in the bottom.

Figure 3 shows the result of the two YGS runs: the distribution is clearly bimodal in the XY-GS analysis (Figure 3A) and unimodal in the ZW-GS (Figure 3B). This strongly suggests that *L. longipalpis* s.l. has a XY sex determination system, which we experimentally confirmed as follows. To avoid the possible interference of contaminants (*e.g.*, a sex-specific bacteria), we first sought among the 1539 scaffolds those that have BlastX hit against an Arthropod protein; we found 35 scaffolds. We then picked at random six candidates to test with PCR using male and female DNA to confirm sex linkage (Supplementary Table S1). All six candidates were amplified only in males (Figure 4) confirming that *L. longipalpis* s.l. has a XY/XX sex determination system.

In total, 1539 scaffolds (3.8% of the total) with 1,212,666 bp (0.7%) altogether had more than 70% unmatched *k*-mers (XY-GS), and are suspect of being Y-linked in *L. longipalpis* s.l. This amount of sequence is comparable with other species (*e.g.*, *D. virilis*, Supplementary Table S2). These values should be seen as rough and probably sub-estimates: Y and W chromosomes frequently have a lot of repetitive sequences which fail to assemble.

Although there is no sign of a W-linked sequences such as a bimodal distribution when using male reads, we also looked at the scaffolds in the right tail of Figure 3B. There are 223 scaffolds with more than 70% unmatched *k-mers* (ZW-GS), containing 161,858 bp. Seven of them had a BlastX hit with an Arthropod using our Blobtools database, but when we manually checked them using NCBI BlastX, they are repetitive sequences such as transposable elements. Repetitive sequences are prone to misassemblies that can generate spurious YGS signals (Carvalho and Clark 2013).

Given that we have identified a set of Y-linked scaffolds, we performed a preliminary search for a sex determining gene. We looked at scaffolds with more that 70% unmatched *k-mers* (XY-GS) with BlastX hit with Arthropod as they would be more likely to contain a sex determining gene. The candidates from the 35 scaffolds were manually annotated. Upon this closer analysis, they were found to be either spurious hits, transposons, or had early stop codons or only represented a small part of the gene sequence, suggesting (in the latter two cases) that they are pseudogenes. It is important to keep in mind that our preliminary analysis would not be able to find the sex determining gene if it is noncoding RNA or a fast-evolving protein, as is the case in several other insects (Kiuchi *et al.* 2014; Hall *et al.* 2015). The identification of the Muller element from the candidates with a *D. melanogaster* ortholog did not show any clear pattern that would had allowed us to determine the Muller element that corresponds to the sex chromosome in *L. longipalpis* s.l. (Supplementary Table S2).

## Discussion

The sex chromosome system is an important trait as it will influence many aspects of genome evolution. Identifying the system (XY/XX or ZW/ZZ) is difficult when there are no morphological differences between the sex chromosomes, or when live samples are not available. Here, we applied the YGS method (Carvalho and Clark 2013) to identify the sex chromosome system (XY or ZW) in the sand fly *L. longipalpis* s.l., which is a challenging case due to its homomorphic sex chromosomes. The computational results clearly indicated a XY system (Figure 3), which was experimentally confirmed by PCR (Figure 4). Hence YGS is a reliable tool for the identification of the sex determination system. This conclusion is strengthened by the finding that the known ZW species *T. californica* produced the inverse pattern when compared with *L. longipalpis*, confirming that the YGS method can detect a ZW system (Supplementary Figure S1). Readers interested in applying the approach we describe may benefit from the practical guidance we presented in Supplementary File S2.

The primary purpose of the present work was to identify the sex-determination system, but we also did a preliminary (and unsuccessful) attempt of identification of the sex-determination gene by looking at protein-coding genes is the candidate Y-linked scaffolds. This failure is quite expected: Identifying the sex-determining gene by sequence similarity is a difficult task since it varies among Diptera families, and is unknown in many of them ( Saccone *et al.* 2011; Salz 2011; Petrella *et al.* 2019). None of the candidate protein-coding genes that were identified on the Y seem good candidates for the sex-determining gene, as they either had a premature stop codon or were otherwise incomplete genes. The *L. longipalpis* s.l. genome seems to be somewhat difficult to assemble, as judged by the fragmentation of our assembly and by the number of missing genes in the VectorBase assembly (Table 2). Hence, it is possible that some of the incomplete genes we found in the Y-linked contigs represent full-length genes, which were not fully assembled, or that the gene was lost altogether during the assembly. Our scan for candidates of a sex-determining gene was only preliminary and would not be able to identify poorly conserved or novel genes, as occurs in some mosquitoes and medfly (Hall *et al.* 2015; Krzywinska *et al.* 2016; Meccariello *et al.* 2019), if there are multiple copies of the gene and/or a similar paralog or if it might be located in an autosome as it can happen in *Musca domestica* (Hamm *et al.* 2014; Sharma *et al.* 2017), or even noncoding RNA genes as in *Bombyx mori* (Katsuma *et al.* 2018). Another possible explanation for the lack of candidates is that, although *L. longipalpis* s.l. has XY system, it might not have a M-factor, as happens in *Drosophila* (Salz 2011). Further work will be needed to uncover the sex determination pathway in sandfly. This effort would greatly benefit from a more contiguous assembly.

Nonetheless, besides the identification of the sex-chromosome system, our analysis provides the first description of male-specific markers in *L. longipalpis* s.l. Considering that *L. longipalpis* s.l. is a species complex (Araki *et al.* 2009; Vigoder *et al.* 2015; Souza *et al.* 2017) the use of such markers may offer information about the evolution of the *L. longip*alpis complex, such the role of sex-chromosomes as barriers to gene flow (*e.g.*, Fontaine *et al.* 2015). Also, the identification of male-specific genetic markers may provide an important tool for the development of insect control strategies such as allowing the production of male-only lines for SIT programs (Meza *et al.* 2014; Achee *et al.* 2019).

Other techniques have been used to identify the sex chromosome system; however, they require RNA sequencing or RAD sequencing (Jeffries *et al.* 2018; Meisel *et al.* 2020). The YGS method was originally designed for species with heteromorphic sex-chromosomes (Carvalho and Clark 2013), and our results show that it also works in a system with homomorphic sex-chromosomes. The expanded use of the YGS method (Carvalho and Clark 2013) to identify the sex chromosome system needs no more than separate Illumina DNA sequencing of males and females. It is a simple and reliable method for identification of the heterogametic sex (and of sequences from the Y or W chromosomes), and it is especially useful in species with homomorphic sex chromosomes or when fresh specimens are required for cytogenetic studies are not readily available.
